# Assessing Hospital Data Quality: Application of a Data Quality Tool in 15 Countries

**DOI:** 10.23889/ijpds.v9i5.2889

**Published:** 2024-09-18

**Authors:** Namneet Sandhu, Sarah Whittle, Lucia Otero Varela, Cathy A Eastwood, Danielle A Southern, Hude Quan

**Affiliations:** 1 University of Calgary

## Introduction

Globally, differences in coding practices and guidelines lead to variations in hospital-administrative data. Studies on patient safety found that better hospital-data quality is associated with higher rates of patient safety events. This study outlines our process of creating a standardized tool for countries to assess their hospital-administrative data quality.

## Approach

We developed 24 indicators across five different dimensions through a Delphi-consensus method. To test applicability of these indicators, we approached representatives from 50 countries with an online survey comprised of qualitative and quantitative questions. Characteristics of each country and indicator scores were noted. A data quality overall score, out of 20, was calculated for each country based on survey responses. The score was classified into 3 data quality categories: high (≥18), moderate (13-17.9), and low (<13).

## Results

Of the 50 countries invited, 17 responded. Surveys from two countries were excluded due to insufficient data. Country responses (n=15) were evaluated and scored by dimension. The data quality indicators showed positive face validity and were applicable for most countries providing comparative information for development of the tool with good discrimination. Canada, USA, New Zealand, UK, and Spain were among the countries with an overall high data quality score (≥18). Most countries scored high in 3 out of 5 dimensions of data quality. A few countries scored 0 out of 4 in ‘Relevance’ and ‘Timeliness’ dimensions resulting in a lower overall score.

## Conclusion

We propose our developed tool be used for comparing hospital-administrative data quality for applying the same standard across countries.

